# Stress-Induced Immunosuppression Inhibits Regional Immune Responses in Chicken Adipose Tissue Partially through Suppressing T Cells by Up-Regulating Steroid Metabolism

**DOI:** 10.3390/ani14020225

**Published:** 2024-01-10

**Authors:** Wei Zhang, Xinxin Xu, Rui Zhang, Yufei Tian, Xiaoli Ma, Xiangnan Wang, Yi Jiang, Chaolai Man

**Affiliations:** College of Life Science and Technology, Harbin Normal University, Harbin 150025, China; 18946033862@163.com (W.Z.); 15049723002@163.com (X.X.); 18845647812@163.com (R.Z.); tyf_smhz@163.com (Y.T.); maxiaoli62@163.com (X.M.); wangxiangnan0513@163.com (X.W.); 16664584444@163.com (Y.J.)

**Keywords:** chicken, lipid metabolism, stress-induced immunosuppression, adipose tissue, immune response

## Abstract

**Simple Summary:**

Adipose tissue (AT) plays an important role in maintaining lipid homeostasis and regulating immune function in mammals. However, the regulatory functions and mechanisms of avian AT in immune response have not been reported. In this study, we found that chicken AT actively responded to stress-induced immunosuppression (SIIS), and adipose triglyceride lipase (*ATGL*), carnitine palmitoyltransferase 1A (*CPT1A*), and 3-hydroxy-3-methylglutaryl-CoA reductase (*HMGCR*) all constitute the key genes involved in the processes of SIIS inhibiting the immune response to the Newcastle disease virus vaccine. Moreover, SIIS significantly inhibited the different phases of the primary and secondary immune response in AT through the upregulation of steroid anabolism. Interestingly, AT could inhibit or promote immune responses through the upregulation or downregulation of steroid metabolism, respectively. The miR-29a/c-3p-*HMGCR* network is a potential regulation mechanism of AT participating in immune response and circulating miR-29a/c-3p is a potential molecular marker. This study can provide references for further investigating the immune functions of AT.

**Abstract:**

Lipid metabolism plays an important role in maintaining lipid homeostasis and regulating immune functions. However, the regulations and mechanisms of lipid metabolism on the regional immune function of avian adipose tissue (AT) have not been reported. In this study, qRT-PCR was used to investigate the changes and relationships of different lipid metabolism pathways in chicken AT during stress-induced immunosuppression (SIIS) inhibiting immune response to Newcastle disease virus vaccine, then the miRNA regulation patterns of 3-hydroxy-3-methylglutaryl-CoA reductase (*HMGCR*) gene and its potential applications were further identified. The results showed that AT actively responded to SIIS, and *ATGL*, *CPT1A* and *HMGCR* were all the key genes involved in the processes of SIIS inhibiting the immune responses. SIIS significantly inhibited the natural and specific immune phases of the primary immune response and the initiation phase of the secondary immune response in AT by suppressing T cells by up-regulating steroid anabolism. Moreover, steroid metabolism could play dual roles in regulating the regional immune functions of AT. The miR-29a/c-3p-*HMGCR* network was a potential regulation mechanism of steroid metabolism in AT, and serum circulating miR-29a/c-3p had the potential as molecular markers. The study can provide valuable references for an in-depth investigation of the regional immune functions regulated by lipid metabolism in AT.

## 1. Introduction

Adipose tissue (AT) is the intersection linking immunity and metabolism [1,2], as immune-related cells in AT play key roles in regulating the body’s immune homeostasis and immune protection [3,4], and change in lipid metabolism of AT is an important pathway affecting immune functions [5,6]. In addition, lymphocytes in AT mainly utilize fatty acids to maintain their biological functions [7,8,9,10]; for example, resident memory T (T_RM_) cells increase the uptake of extracellular free fatty acids (FFAs) by up-regulating the expressions of fatty acid binding protein 4 (FABP4) and fatty acid binding protein 5 (FABP5) to maintain their lifespans and functions [11]. Recently, it has been shown that the change in lipid metabolism of the tumor microenvironment (TME) is an important mode affecting the functions and activities of infiltrating lymphocytes, which has become a research hotspot in the field of tumor prevention and treatment [12,13]. It is worth mentioning that the low efficiency of capillary circulation in white adipose tissue (WAT) [14], which can cause the paracrine effect of adipose tissue cells to form a certain regional microenvironment state, so changes in lipid metabolisms of AT can inevitably affect the regional immune functions and states of WAT. However, the effects of lipid metabolism on regional immune functions in avian AT have not been reported.

AT is widely distributed in the body and links different tissues and organs [15]. Studying the regulations and mechanisms of changes in lipid metabolism on the regional immune functions of AT is of positive research value in many aspects, such as the relationships between regional immunity of AT and systemic immunity, improvement of the body’s immune function, and potential development and application of AT. Chickens are in the middle position of evolution and are important model animals for studying immune functions [16]. In this study, Hy-line Gray chickens were selected as model animals, and SIIS and immune response of chickens were induced by dexamethasone (Dex) and Newcastle disease virus (NDV) attenuated vaccine, respectively. Then, we monitored the dynamic changes of different lipid metabolic pathways in WAT at different candidate time points during SIIS, inhibiting the primary and secondary immune responses to the NDV vaccine. Finally, we further analyzed the regulatory patterns between *HMGCR* (a key enzyme gene of cholesterol metabolism) and miR-29a/c-3p and identified the potential applications of the candidate miRNAs as circulating biomarkers. This study can provide valuable references for an in-depth investigation of the immunomodulatory functions of lipid metabolism in AT.

## 2. Materials and Methods

### 2.1. Ethics Statement

All animal experiments were carried out under the guidelines approved by the Institutional Animal Care and Use Committee (IACUC). The proposed study protocols were approved by the Laboratory Animal Care Committee of Harbin Normal University (No. HNUARIA2021001).

### 2.2. Experimental Grouping and Sample Collection

The experiment was based on the previous animal treatments in our lab [17]. Three hundred and sixty healthy unvaccinated 1-day-old Hy-line Gray chickens from Harbin Xiangfang hatchery were randomly divided into four groups: control group, Dex group (dexamethasone treatment group), ND group (NDV attenuated vaccine immunization group) and Dex + ND group (dexamethasone and NDV vaccine dual-factor treatment group), with 3 replicates per group and 30 chickens per replicate, and reared in isolation (The room temperature of 1–7-day-old chickens was 35 °C, the room temperature of 7–42 day-old chickens was 27 °C, and the humidity was 50–70%) and fed ad libitum (feed formulation: vitamins (V_A_, V_b3_, V_E_, V_K_, V_B6_, V_B12_), fishmeal, corn, high-quality soybean meal, acid preparations, calcium propionate, calcium hydrogen phosphate, rock flour, copper sulfate, ferrous sulfate, manganese sulfate, zinc oxide, salt, lysine, methionine, sodium selenite, etc.). Chickens from the Dex group and the Dex + ND group were treated with dexamethasone (Dex) (1.5 mg/kg, purchased from China Huaxu Veterinary Pharmaceuticals Ltd.) for three days during 5–7 days of age and 19–21 days of age, respectively. Chickens from ND group and Dex + ND group were vaccinated with 0.05 mL NDV LaSota strain (the viral content was not less than 10^6.0^EID_50_ per feather portion) (Harbin Veterinary Research Institute, Harbin, China) by eye-drop at 7 days old and boosted at 21 days old, respectively, while chickens from the control group and the Dex group were treated with vaccine diluted salt solution (Harbin Veterinary Research Institute, Harbin, China). For the convenience of comparison, the sampling time points of the four treatment groups were the same, and they all adopted the same labeling method (days post-immunization (dpi)). At 2, 5, 7, 14 and 21 days post the primary and secondary immunization, three chickens were randomly selected from each replicate of each group, and thymus, bursa, spleen, abdominal adipose tissue (AAT) and serum were collected and stored at −80 °C.

### 2.3. Detection of Antibody Titer and Analysis of Organ Coefficient

Antibody levels and organ coefficients were detected according to the methods of previous studies [17,18]. Serum NDV antibody titers were detected by hemagglutination (HA) and hemagglutination inhibition (HI) assays using standard antigens of the NDV LaSota strain (Harbin Veterinary Medical Research Institute, Harbin, China) and expressed as the reciprocal of log2 values [17,18,19]. The body weight, thymus, bursa and spleen from the nine randomly selected chickens at each time point were used to analyze the body weight coefficients and organ coefficients, respectively.

### 2.4. Reverse Transcription and Quantitative Real-Time Fluorescence PCR (qRT-PCR)

Total RNA was extracted from AT and serum and quantified (300 ng/μL RNA of each sample), respectively. Reverse transcription was performed using the FSQ-301 kit (TOYOBO, Shanghai, China) according to the manufacturer’s instructions. QRT-PCR system for mRNAs was 10 μL, containing 0.6 μL cDNA, 0.3 μL of each primer, 5 μL 2 × SYBR Green I (TOYOBO, Shanghai, China), 0.2 μL 50 × ROX (TOYOBO, Shanghai, China), 3.6 μL RNA-free water. QRT-PCR system for miRNA was 10 μL, including 0.8 μL cDNA, 0.3 μL of each primer, 5 μL 2 × SYBR Green I (TOYOBO, Shanghai, China) 0.2 μL 50 × ROX (TOYOBO, Shanghai, China), 3.4 μL RNA-free water. QRT-PCR procedures of the mRNAs and miRNAs were: 95 °C for 1 min, followed by 45 cycles, 95 °C for 15 s, 60 °C for 30 s, 72 °C for 30 s, and 72 °C final extension for 30 s. The *β-actin* gene and *U6* were selected as the internal references mRNAs and miRNAs, respectively. All primer sequences are provided in Table 1.

### 2.5. Statistical Analysis

The 2^−ΔΔCt^ method was used to calculate the relative expression activities of target genes and miRNAs [20]. All data were presented as meaning ± SD from at least 3 sets of independent experiments. Data between different treatment groups were analyzed using an independent *t*-test, and data within the same treatment group were analyzed using ANOVA. Independent *t*-tests and ANOVA were performed using SPSS 20.0 software (https://www.ibm.com/products/spss-statistics, accessed on 15 June 2023) and GraphPad Prism _v8.2.1 software (https://www.graphpad.com/scientific-software/prism/, accessed on 17 June 2023) was used to plot, *p* < 0.05 indicated statistical significance.

## 3. Results

### 3.1. Analysis of Organ Coefficients and Serum Antibodies

**Figure 1:**
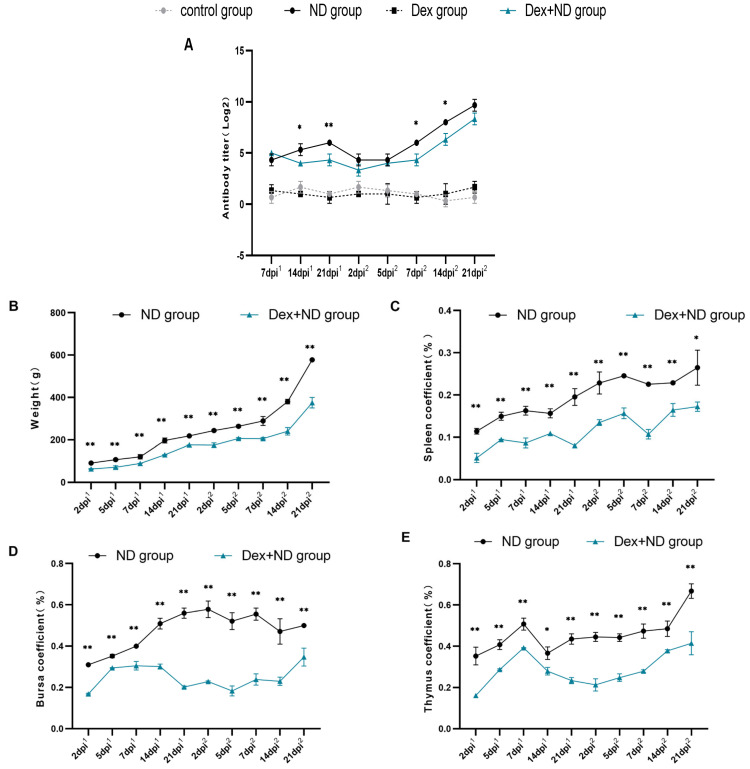
Identifications of immune response and Dex-induced immunosuppressed chicken models. (**A**) NDV antibody levels in different treatment groups; (**B**) body weight coefficients of the ND group and Dex + ND group; (**C**–**E**) organ coefficients of spleen, bursa and thymus of ND group and Dex + ND group, respectively. The horizontal coordinate dpi^1^ represented the days post-primary immunization, and dpi^2^ represented the days post-secondary immunization. * represented a significant difference between the ND group and Dex + ND group at the same time point (*p* < 0.05), ** represented a highly significant difference between the ND group and Dex + ND group at the same time point (*p* < 0.01).

HA-HI analysis showed that the NDV antibody levels in the ND group showed a gradual increase in the primary immunity (7–21 dpi^1^) and peaked at 21 dpi^1^; in the secondary immunity (2–21 dpi^2^), the NDV antibody levels showed a continuous increase, and the peak level was significantly higher than that of the primary immunity. In comparison between the ND group and Dex + ND group, the changing trend of NDV antibody of the Dex + ND group was similar to that of the ND group, but the antibody levels were significantly lower than those of the ND group, and there were significant differences at several time points (14 dpi^1^, 21 dpi^1^, 7 dpi^2^ and 14 dpi^2^) (*p* < 0.05) (Figure 1A). In addition, comparing the body weight coefficients and three organ coefficients, including thymus, bursa and spleen, between the ND group and Dex + ND group, the body weight coefficients and all the organ coefficients of the Dex + ND group were significantly lower than those of ND group (*p* < 0.05) (Figure 1B–E). These results showed that the primary and secondary immune response chickens and stress-induced immunosuppressed chickens were successfully established, respectively [17].

### 3.2. Expression Characteristics of Adipose Tissue ATGL, CPT1A and HMGCR Genes in the State of SIIS

**Figure 2:**
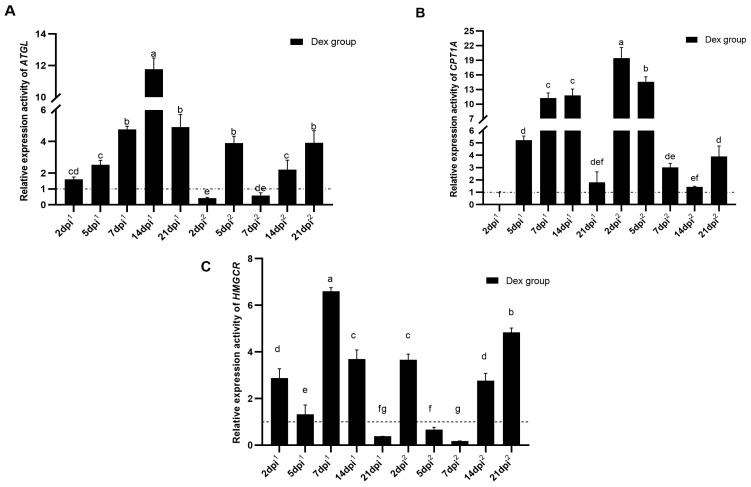
Relative expression activities of adipose tissue *ATGL*, *CPT1A* and *HMGCR* genes in Dex group. (**A**) The expression activity of *the ATGL* gene in the Dex group; (**B**) The expression activity of *the CPT1A* gene in the Dex group; (**C**) The expression activity of the *HMGCR* gene in the Dex group. The horizontal coordinate dpi^1^ represented the days post-primary immunization, and dpi^2^ represented the days post-secondary immunization. The horizontal dashed line corresponded to the relative expression activity of 1. Different letters above the bars indicated significant differences between different time points (*p* < 0.05), and the same letters indicated non-significant differences (*p* > 0.05).

To explore whether the lipid metabolism of chicken AT positively responds to the process of SIIS, we analyzed the expression characteristics of adipose tissue *ATGL*, *CPT1A* and *HMGCR* genes in the Dex group. For ease of comparison, we artificially classified the expression activities of all the candidate targets in this study into three levels: over 2 for high expression, less than 1 for low expression, and medium expression between 1 and 2. QRT-PCR results showed that *ATGL* gene expression activities changed significantly among different time points (*p* < 0.05) (Figure 2A). During the process of the primary SIIS, *ATGL* genes were all highly expressed except for the moderate expression on 2 dpi^1^; in the process of the secondary SIIS, *ATGL* was highly expressed on 5 dpi^2^, 14 dpi^2^ and 21 dpi^2^, and lowly expressed on 2 dpi^2^ and 7 dpi^2^. In addition, *CPT1A* gene expression activities changed significantly among different time points (*p* < 0.05) (Figure 2B). During the primary SIIS, *CPT1A* gene was highly expressed on 5 dpi^1^, 7 dpi^1^ and 14 dpi^1^, moderately expressed on 21 dpi^1^, and lowly expressed on 2 dpi^1^; in the process of secondary SIIS, *CPT1A* gene was highly expressed on 2 dpi^2^, 5 dpi^2^, 7 dpi^2^ and 21 dpi^2^, and moderately expressed on 14 dpi^2^. Additionally, *HMGCR* gene expression activities changed dramatically during the primary and secondary SIIS (*p* < 0.05) (Figure 2C). For example, *HMGCR* was highly expressed on 2 dpi^1^, 7 dpi^1^ and 14 dpi^1^, moderately expressed on 5 dpi^1^ and lowly expressed on 21 dpi^1^ during the primary SIIS; and *HMGCR* was highly expressed on 2 dpi^2^, 14 dpi^2^ and 21 dpi^2^ and lowly expressed on 5 dpi^2^ and 7 dpi^2^ during the secondary SIIS.

Overall, during the primary SIIS, *ATGL*, *CPT1A* and *HMGCR* genes were all up-regulated as their common features (except for *CPT1A* on 2 dpi^1^ and *HMGCR* on 21 dpi^1^), but the change trends of *ATGL* and *CPT1A* were similar, which were certainly different from that of *HMGCR*; during the secondary SIIS, *ATGL*, *CPT1A* and *HMGCR* genes were all up-regulated as their common features (except for *ATGL* on 2 and 7 dpi^2^ and *HMGCR* on 5 and 7 dpi^2^), and there were similarities between *CPT1A* and *HMGCR* in terms of change trends, while there were some differences between them and *ATGL*.

### 3.3. Expression Characteristics of Adipose Tissue ATGL, CPT1A and HMGCR Genes in the Processes of SIIS Inhibiting Immune Responses

**Figure 3:**
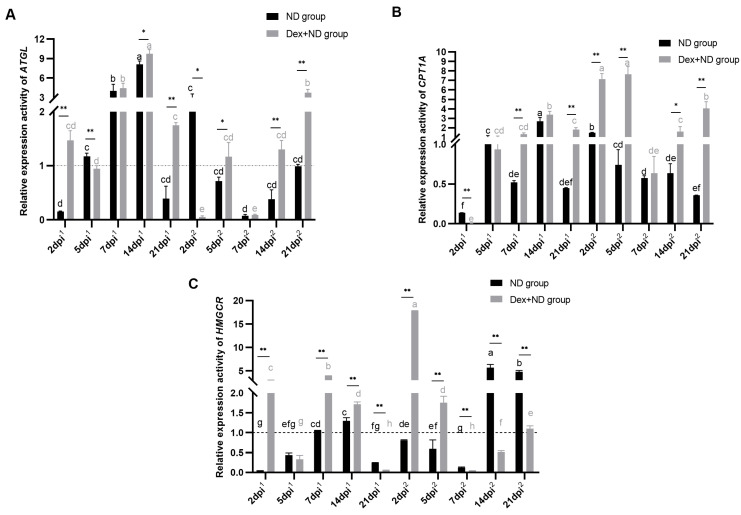
Relative expression activities of adipose tissue *ATGL*, *CPT1A* and *HMGCR* genes in the ND group and Dex + ND group. (**A**) Expression activity of *ATGL* gene in the ND group and Dex + ND group; (**B**) Expression activity of the *CPT1A* gene in the ND group and Dex + ND group; (**C**) Expression activity of the *HMGCR* gene in the ND group and Dex + ND group. The horizontal dashed line corresponded to a relative expression activity of 1. The horizontal coordinate dpi^1^ represented the days post the primary immunization, and dpi^2^ represented the days post the primary immunization. Different letters on the bars in the same group indicated significant differences among different time points (*p* < 0.05). * represented a significant difference between the ND group and Dex + ND group at the same time point (*p* < 0.05), ** represented a highly significant difference between the ND group and Dex + ND group at the same time point (*p* < 0.01).

QRT-PCR results showed that, during the primary immune response, the *ATGL* gene was highly expressed on 7 dpi^1^ and 14 dpi^1^, moderately expressed on 5 dpi^1^, and lowly expressed on 2 dpi^1^ and 21 dpi^1^; during the secondary immune response, the *ATGL* gene was highly expressed on 2 dpi^2^, moderately expressed on 21 dpi^2^, and lowly expressed on 5 dpi^2^, 7 dpi^2^ and 14 dpi^2^ (Figure 3A). Significant changes in *CPT1A* gene expression activities were also found (Figure 3B), for example, during the primary immune response of ND group, *CPT1A* was highly expressed on 14 dpi^1^, moderately expressed on 5 dpi^1^, and lowly expressed on 2 dpi^1^, 7 dpi^1^ and 21 dpi^1^; during the secondary immune response, *CPT1A* was all lowly expressed except for 2 dpi^2^ with moderate expression. According to the comparisons between the ND group and Dex + ND group, the expression activities of the *CPT1A* gene showed significant differences at multiple time points, such as 2 dpi^1^, 7 dpi^1^, 21 dpi^1^, 2 dpi^2^, 5 dpi^2^, 14 dpi^2^ and 21 dpi^2^. QRT-PCR results showed that, in the ND group, *HMGCR* was moderately expressed on 7 and 14 dpi^1^ and lowly expressed on 2, 5 and 21 dpi^1^ during the primary immune response; during the secondary immune response, *HMGCR* was highly expressed on 14 and 21 dpi^2^, while lowly expressed on 2, 5 and 7 dpi^2^. Compared to the ND group, *HMGCR* was significantly up-regulated at multiple candidate time points, such as 2 dpi^1^, 7 dpi^1^, 14 dpi^1^, 2 dpi^2^ and 5 dpi^2^ in the Dex + ND group (*p* < 0.01), but significantly down-regulated at the other time points (*p* < 0.01). Moreover, the differences were highly significant between the two groups at all the candidate time points (except 5 dpi^1^) (*p* < 0.01).

Overall, in the primary immune response, *CPT1A* and *HMGCR* genes were mainly down-regulated, and *ATGL* was mainly up-regulated in the ND group, while *ATGL*, *CPT1A* and *HMGCR* genes were mainly up-regulated as their common features in the Dex + ND group. In the secondary immune response, *ATGL*, *CPT1A* and *HMGCR* genes were mainly down-regulated in the ND group, while they were mainly up-regulated in the Dex + ND group as their common features. So, the common feature between the ND group and Dex + ND group was mainly characterized by the significant differences (*p* < 0.01) at the most candidate time points during the primary and secondary immune responses.

### 3.4. Identification of Adipose Tissue T Cells Involving in the Processes of SIIS Inhibiting Immune Responses

**Figure 4:**
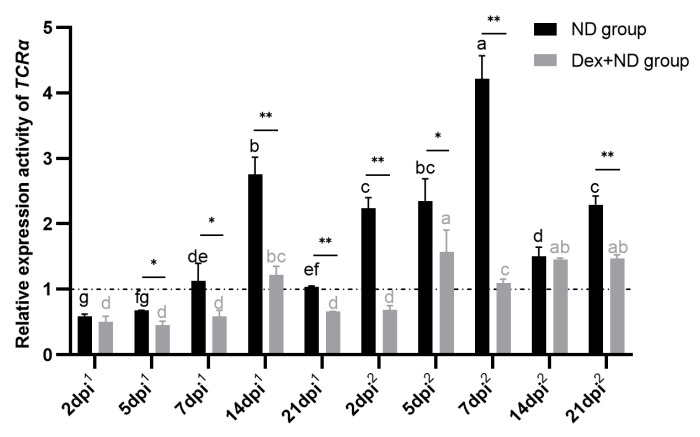
Relative expression activity of adipose tissue *TCRα* gene in ND group and Dex + ND group. The horizontal dashed line corresponded to a relative expression activity of 1. The horizontal coordinate dpi^1^ represented the days post the primary immunization, and dpi^2^ represented the days post the secondary immunization. Different letters on the bars in the same group indicated significant differences between different time points (*p* < 0.05). * represented a significant difference between the ND group and Dex + ND group at the same time point (*p* < 0.05), ** represented a highly significant difference between the ND group and Dex + ND group at the same time point (*p* < 0.01).

To further investigate how SIIS inhibits the regional immune responses of AT, we selected and analyzed the expression activity of the *TCRα* gene between the ND group and the Dex + ND group. QRT-PCR results showed that *TCRα* gene expression activities were significantly changed (*p* < 0.05) (Figure 4). In the ND group, during the primary immune response, *TCRα* was highly expressed on 14 dpi^1^, moderately expressed on 7 dpi^1^ and 21 dpi^1^, and lowly expressed at the other time points; during the secondary immune response, *TCRα* was highly expressed on 2 dpi^2^, 5 dpi^2^, 7 dpi^2^ and 21 dpi^2^, and moderately expressed on 14 dpi^2^. In comparison between the ND group and Dex + ND group, *TCRα* gene expression activities were significantly different (*p* < 0.05) at the most candidate time points in the processes of the primary and secondary immune responses. Moreover, the expression activities of the *TCRα* gene in the Dex + ND group were all lower than those in the ND group.

### 3.5. Identification of Game Relationship between miR-29a/c-3p and HMGCR Gene in Adipose Tissue during SIIS Inhibiting Immune Responses

**Figure 5:**
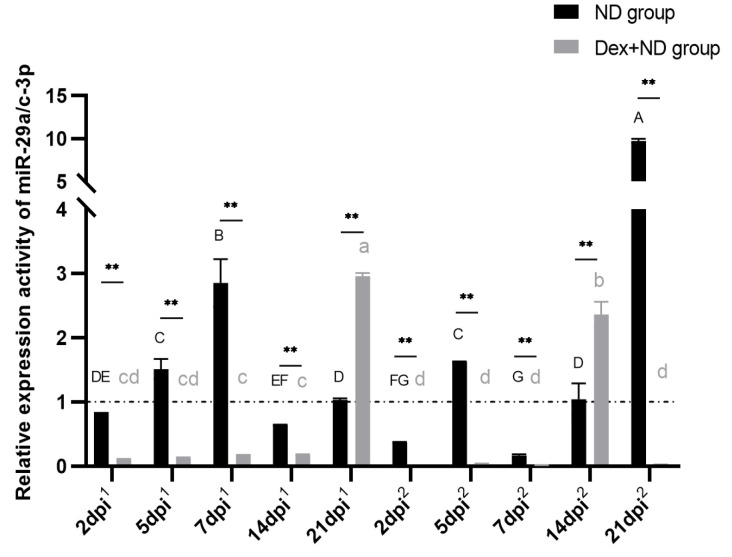
Relative expression activity of adipose tissue miR-29a/c-3p in ND group and Dex + ND group. The horizontal dashed line corresponded to a relative expression activity of 1. The horizontal coordinate dpi^1^ represented the days post the primary immunization, and dpi^2^ represented the days post the secondary immunization. Different letters on the bars in the same group indicated significant differences between the same time points (*p* < 0.05). ** represented a highly significant difference between the ND group and Dex + ND group at the same time point (*p* < 0.01).

*HMGCR* is a key enzyme gene involved in the steroid metabolic pathway, and steroid lipids play key roles in the regulation of immune functions. Therefore, we further analyzed the expression characteristics of adipose tissues miR-29a/c-3p between the ND group and Dex + ND group in order to explore the regulation pattern of adipose tissue *HMGCR* gene in vivo. It is shown that both miR-29a-3p and miR-29c-3p can target the *HMGCR* gene [21], and they have highly homologous sequences [21,22], and the stem-loop primers cannot distinguish them. Therefore, the stem-loop qRT-PCR can reflect their common quantification results of miR-29a-3p and miR-29c-3p. The results showed that there were highly significant differences in the expression activities of adipose tissue miR-29a/c-3p between the ND group and Dex + ND group at the candidate time points (*p* < 0.05) (Figure 5). In the ND group, miR-29a/c-3p were highly expressed on 7 dpi^1^, moderately expressed on 5 dpi^1^ and 21 dpi^1^, and lowly expressed on 2 dpi^1^ and 14 dpi^1^ during the primary immune response; in the process of secondary immune response, miR-29a/c-3p were highly expressed on 21 dpi^2^, moderately expressed on 5 dpi^2^ and 14 dpi^2^, and lowly expressed on 2 dpi^2^ and 7 dpi^2^. In Dex + ND group, miR-29a/c-3p were highly expressed on 21 dpi^1^ and lowly expressed on 2 dpi^1^, 5 dpi^1^, 7 dpi^1^ and 14 dpi^1^ during the primary immune response; during the secondary immune response, and miR-29a/c-3p were only highly expressed on 14 dpi^2^, and lowly expressed at other time points. Interestingly, the differences in expression activities of adipose tissue miR-29a/c-3p were highly significant at all the candidate time points between the ND group and the Dex + ND group (*p* < 0.01).

**Figure 6:**
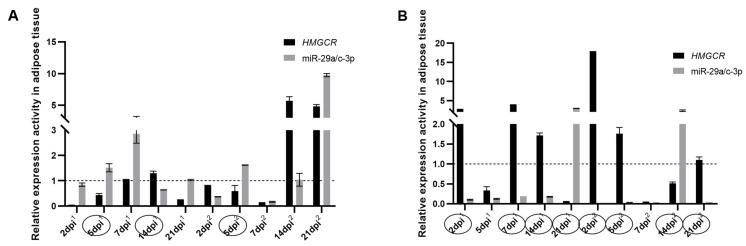
Game relationships between miR-29a/c-3p and *HMGCR* gene in adipose tissue. (**A**) Game relationships between miR-29a/c-3p and *HMGCR* gene in ND group. (**B**) Game relationships between miR-29a/c-3p and *HMGCR* gene in Dex + ND group. The horizontal dashed line corresponded to a relative expression activity of 1. The horizontal coordinate dpi^1^ represented the days post the primary immunization, and dpi^2^ represented the days post the secondary immunization. Circles indicated time points with game relationships between miR-29a/c-3p and the *HMGCR* gene.

To identify the molecular mechanisms underlying the regulation of adipose tissue *HMGCR* gene expression, we compared and analyzed the game relationships of expression activities between miR-29a/c-3p and the *HMGCR* gene in AT. The results showed that miR-29a/c-3p and *HMGCR* genes had significant game relationships between them at multiple candidate time points. For example, in ND group, miR-29a/c-3p and *HMGCR* gene expression activities were gamely regulated (miR-29a/c-3p up-regulated expression and *HMGCR* down-regulated expression, or miR-29a/c-3p down-regulated expression and *HMGCR* up-regulated expression) on 5 dpi^1^, 14 dpi^1^ and 5 dpi^2^ (Figure 6A); in Dex + ND group, miR-29a/c-3p and *HMGCR* gene expression activities were gamely regulated at the most candidate time points (except for 5 dpi^1^ and 7 dpi^2^) (Figure 6B).

### 3.6. Identification of Serum Circulating miR-29a/c-3p as Molecular Markers

**Figure 7:**
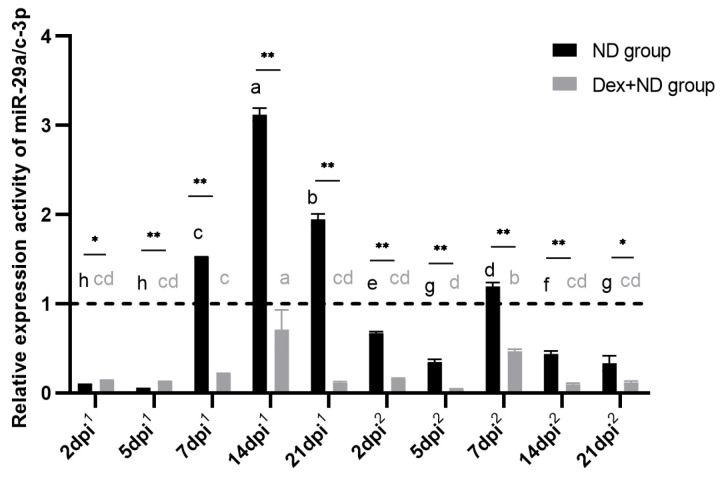
Expression levels of circulating miR-29a/c-3p in ND group and Dex + ND group. The horizontal dashed line corresponded to a relative expression activity of 1. The horizontal coordinate dpi^1^ represented the day after the primary immunization, and dpi^2^ represented the day after the secondary immunization. Different letters on the bars in the same treatment group indicated significant differences among different time points (*p* < 0.05). * represented significant differences between the ND group and Dex + ND group at the same time point (*p* < 0.05), and ** represented highly significant differences between the ND group and Dex + ND group at the same time point (*p* < 0.01).

To investigate the potential application of serum circulating miR-29a/c-3p in SIIS-inhibiting immune responses further, we analyzed the expression levels of serum circulating miR-29a/c-3p in the ND group and Dex + ND group. QRT-PCR results showed that, in the ND group, serum circulating miR-29a/c-3p were up-regulated on 7 dpi^1^, 14 dpi^1^ and 21 dpi^1^ and down-regulated at the other time points during the primary immune response; in the process of secondary immune response, serum circulating miR-29a/c-3p were up-regulated on 7 dpi^2^ and down-regulated at the other time points. Compared to the ND group, serum circulating miR-29a/c-3p were all down-regulated in the Dex + ND group during both immune responses, and there were significant differences at all the candidate time points between the ND group and Dex + ND group (*p* < 0.05), especially on 7 dpi^1^, 14 dpi^1^, 21 dpi^1^ and 7 dpi^2^ (Figure 7).

## 4. Discussion

In recent years, the immunomodulatory function of AT has become a hot topic of research [1,2]. However, studies on the regulation of regional immune function in avian AT by lipid metabolism have not been reported. Previous studies in our lab have shown that there are regional immune responses to the NDV vaccine in chicken AT, and lipid metabolisms of AT actively respond to the NDV secondary immune response through down-regulation [17]. However, the mechanisms of lipid metabolisms involved in the regulation of regional immune function of AT are still unclear. In this study, in order to explore the patterns and mechanisms of lipid metabolism pathways involved in the regional immune responses of AT, we produced chicken models with SIIS inhibiting immune response and analyzed the changes and functions of different lipid metabolism pathways of AT in the processes of SIIS inhibiting immune responses to NDV vaccine.

Dexamethasone treatment is a classical method to simulate SIIS in animals [23,24,25]. In this study, we utilized Dex to simulate stress-induced immunosuppressed chickens and found that the body weight, spleen, bursa, and thymus of the Dex + ND group chickens were significantly lower than those of the ND group at all candidate time points. Moreover, the NDV antibody levels in the Dex + ND group were lower than those in the ND group. These results fully showed that Dex effectively suppressed the primary and secondary immune responses to the NDV vaccine in chickens, and the Dex-induced immunosuppressed chicken models were prepared successfully. In the state of SIIS, we found that *ATGL* expression activities were up-regulated at the most candidate time points, indicating that SIIS promoted adipose tissue triglyceride catabolism, which in turn produced large amounts of fatty acids as raw materials and material basis for further metabolism [26]. To investigate the further pathways of fatty acid metabolism, we analyzed the expression characteristics of *CPT1A* and *HMGCR* genes in the state of SIIS. *CPT1A* is a key enzyme gene in the fatty acid oxidative catabolic pathway, and its mRNA and protein levels are positively correlated [27]. Exploring the transcriptional activities of *CPT1A* can help understand the changes in fatty acid oxidative metabolism under the condition of SIIS. We chose *HMGCR* as the target gene to explore the changes in sterol metabolism of AT because the *HMGCR* is the most important key enzyme gene in the cholesterol synthesis pathway, and its mRNA is positively correlated with protein level [21,28], so analyzing the transcriptional activities of *HMGCR* gene can reflect the states of sterol anabolisms in the process of SIIS [29]. Intriguingly, we found that the expression activities of adipose tissue *CPT1A* gene showed fluctuating up-regulation during the primary SIIS, indicating that the primary process of SIIS was mainly characterized by increased oxidative catabolism of fatty acids in AT. During the secondary SIIS, adipose tissue *CPT1A* gene expression activities were up-regulated at all the candidate time points, indicating that fatty acid oxidative metabolism was still the main biological event. In addition, both primary and secondary SIIS in the Dex group all caused significantly fluctuating changes in steroid metabolisms of AT, suggesting that adipose tissue steroid metabolism was closely associated with SIIS. For example, adipose tissue *HMGCR* gene expression activities were up-regulated at most candidate time points, suggesting that dexamethasone could induce synthesis of sterol lipids of AT to achieve SIIS, which was similar to the up-regulation of *HMGCR* gene expression in the liver followed stress, thus induced hypercholesterolemia [30,31,32].

Since SIIS can significantly change the activities of different lipid metabolisms in AT, we considered that the changed lipid metabolisms would inevitably significantly affect the regional immune responses of AT because of the low circulating efficiency in WAT; thus, the paracrine secretion of AT could form certain regional microenvironment state [14]. ATGL, as a triglyceride hydrolase, has speed-limiting properties in fat mobilization, and *ATGL* mRNA level is positively correlated with its protein level [33]. In our study, during the primary immune response, the expression activities of *ATGL* in the Dex + ND group were significantly higher than those in the ND group except for 5 dpi^1^, suggesting that SIIS could participate in the regional immune response of AT by up-regulating triglyceride catabolism. The expression activities of *CPT1A* in the Dex + ND group were lower than those in the ND group (2–5 dpi^1^) and higher than those in the ND group (7–21 dpi^1^), indicating that SIIS regulated the primary immune response of AT by down-regulation followed by up-regulation of fatty acid oxidative metabolism. Compared with the ND group, the Dex + NDV group showed increased fat hydrolysis during the primary immune response, which produced a large number of fatty acids, thus inducing subsequent up-regulation of fatty acid oxidative catabolism. During the secondary immune response, the expression activities of *ATGL* gene in the Dex + ND group were higher than those in ND group except for 2 dpi^2^, and the *CPT1A* gene expression activities were also higher than those in ND group at all the candidate time points, but fatty acid catabolism was generally down-regulated in ND group, suggesting that SIIS inhibited the secondary immune response of AT through up-regulating fatty acid oxidative catabolism.

The up-regulating *β*-oxidative catabolism of fatty acids in mitochondria produced large amounts of acetyl coenzyme A (Acetyl-CoA) [34]. However, the further metabolic pathway of these Acetyl-CoAs would not all enter the tricarboxylic acid (TCA) cycle to produce ATP because both immune responses of the ND group were characterized by the down-regulation of fatty acid oxidative catabolism. So, we considered that most of these Acetyl-CoAs would enter the steroid anabolic pathway in AT, which in turn produced large amounts of cholesterol and its derivatives. Of note, cholesterol can promote T cell activation and proliferation by regulating cell membrane lipid raft formation and signal transduction [35], and up-regulation of endogenous cholesterol biosynthesis and uptake may be beneficial for T cell function [36]. However, cholesterol can lock the TCR-CD3ζ of T cells in an inactive conformation, thereby inhibiting T lymphocyte activation and proliferation [37], and too much or too little exogenous cholesterol may lead to T cell dysfunction [38]. Therefore, the regulation of lymphocytes by cholesterol and its derivatives is determined by the tissue microenvironment [12,39,40]. In our study, we considered that the expression up-regulation of steroid lipids in AT mainly exerted an inhibitory effect on the regional immune response. The possible reasons were as follows: firstly, AT had a relative regional microenvironment state due to its relatively slow microcirculation [14,41]; secondly, increased mobilization of triacylglycerol in AT (*ATGL* up-regulation) resulted in the production of a large amount of glycerol and fatty acids [42,43], which could be secreted to the outside of adipocytes, thus affecting the microenvironment of AT; thirdly, increased oxidative catabolism of fatty acids (up-regulation of *CPT1A*) led to massive production of Acetyl-CoAs [44], and some acetyl-CoA entered TCA cycle to generate ATP, but most of them would be used as raw materials to further generate cholesterol, including sterol lipids [45], which could also be transported into the interstitial space of AT and further influenced the adipose tissue microenvironment; fourthly, increased steroid lipids could inhibit functions and activities of T lymphocytes [12]; fifthly, increased lipids accumulation in the adipose tissue microenvironment could lead to metabolic crosstalk among surrounding lymphocytes [46] and lymphocytes absorbed excessive free lipids, which in turn affected immune cells functions [12,13]; sixthly, the massive release of lipids from AT would caused microcirculation disorders and obstruction of oxygen transportation in AT [47,48], resulting in TCA cycle metabolic disorders in AT lymphocytes, inhibiting ATP production and thereby affecting lymphocytes activities; seventhly, given that increased lipid concentrations in TME suppressed lymphocytes activities [12,13,49,50], we believed that the biological effect of increased lipid concentrations in AT, represented by steroid lipids, was to suppress the regional immune response of AT. Admittedly, these were our scientific speculations based on existing knowledge, but the specific mechanisms need to be further studied in the future.

Based on the above analyses, we further explored whether steroid metabolic activities changed positively in the processes of SIIS inhibiting the regional immune responses of AT. Interestingly, we found that adipose tissue *HMGCR* gene expression activities were dramatically different between the ND group and Dex + ND group at the most candidate time points, suggesting that SIIS could inhibit the regional immune responses of AT by regulating steroid metabolic pathways. For example, during the primary and secondary immune responses, the *HMGCR* gene was significantly up-regulated at several time points (2 dpi^1^, 7 dpi^1^, 14 dpi^1^, 2 dpi^2^ and 5 dpi^2^) in the Dex + ND group compared to the ND group. Generally, 2 dpi^1^ was the natural immune response phase, 7–14 dpi^1^ corresponded to the acquired immune response phase of primary immunity, and 2–5 dpi^2^ was the rapid response phase of secondary immunity [51], so the up-regulated *HMGCR* gene suggested that SIIS inhibited these key processes of the primary and secondary immune responses by up-regulating steroid anabolism in AT. It was worth noting that 2–5 dpi^2^ was a critical period for the rapid activating and proliferating of T_RM_ cells in AT [52], and our analyses of *TCRα* expression activities in the ND group also revealed the similar significant up-regulation of T cells during 2–7 dpi^2^, implying the presences of T_RM_ cells in chicken AT and their rapid activation and proliferation. Of note, steroid anabolism was significantly up-regulated in the Dex + ND group, suggesting that SIIS could dramatically inhibit the activation and proliferation of T_RM_ cells through up-regulating steroid metabolic pathways. By comparison, the steroid anabolisms in the ND group were down-regulated during 2–5 dpi^2^, resulting in the reduction of lipids concentration (represented by the decrease in sterol lipid synthesis) in the adipose tissue microenvironment, which in turn better responded to and facilitated the regional secondary immune response of AT. The results were consistent with our lab’s previous findings [17]. Interestingly, we found that the activities of steroid anabolism in the ND group were higher than those in the Dex + ND group during the late stages of the primary (21 dpi^1^) and secondary immune responses (7–21 dpi^2^), and the increased steroid lipids inhibited immune activity [12], which might avoid immune damage caused by strong immune response, suggesting that immune responses also could modulate the intensity of regional immune response through changing steroid metabolic pathway in AT.

To further prove the effects of changed lipid metabolism on the regional immune response of AT, we selected and analyzed the expression activities of the T lymphocyte marker *TCRα* gene. Studies show that most T cells express *TCRα* on their surface, and the mRNA level of *TCRα* is positively correlated with its protein level [53,54,55], and analyzing the expression activities of the *TCRα* gene can reflect the changes in the number of T lymphocytes in AT. We found that the *TCRα* gene expression activities in the Dex + ND group at the most candidate time points were significantly lower than those in the ND group during the primary and secondary immune responses, which fully demonstrated that SIIS could significantly inhibit the numbers and functions of adipose tissue T lymphocytes through up-regulating steroid anabolism. The results also indirectly indicated that a large number of T cells participated in the regional immune response of chicken AT. Of note, in the ND group, the expression activities of the *TCRα* gene in the secondary immunity were higher than those in the primary immunity, which possibly was related to the T_RM_ cells, further indicating the existence of T_RM_ cells in chicken AT. In short, we found that SIIS could inhibit the different phases of primary and secondary regional immune responses of AT through up-regulating steroid anabolism. In addition, AT could suppress the intensity of immune response through up-regulating steroid anabolism during the completion stages of immune responses in the ND group. So, the steroid metabolic pathway played “dual roles” in regulating regional immune functions of AT (up-regulation suppressed immunity and down-regulation promoted immunity).

As a key enzyme of the steroid anabolic pathway, *HMGCR* was a key factor involved in the regulation of the regional immune function of AT [29,56,57], so exploring the molecular mechanism of *HMGCR* expression regulation had an important research necessity to gain insight into the immune functional properties of AT. We found that miR-29a/c-3p were significantly differentially expressed among different treatment groups, indicating that miR-29a/c-3p were key factors involved in the regulation of cholesterol metabolism of AT. Furthermore, we found significant game relationships between miR-29a/c-3p and *the HMGCR* gene at multiple candidate time points in different treatment groups, suggesting that the miR-29a/c-3-*HMGCR* regulatory network was a potential mechanism of cholesterol metabolism in AT. Interestingly, miR-29a/c-3p in the Dex + ND group had significant game relationships with *HMGCR* at more candidate time points compared to the ND group, implying that the miR-29a/c-3-*HMGCR* regulatory network was more sensitive to SIIS, and was possibly a key mechanism by which SIIS inhibited regional immune responses of AT. Of course, there were many miRNAs involved in *HMGCR* regulation, so the specific mechanisms need to be further investigated.

To further identify the potential of circulating miR-29a/c-3p as molecular markers in SIIS affecting immune responses, we found that serum circulating miR-29a/c-3p expression levels were significantly down-regulated in the Dex + ND group at the most candidate time points compared to ND group, suggesting that SIIS might inhibit immune responses by down-regulating circulating miR-29a/c-3p expression levels and also implying the potential value of serum circulating miR-29a/c-3p as molecular markers in the process of detecting the effect of SIIS on immune responses.

## 5. Conclusions

We first found that SIIS can inhibit the regional immune response of chicken adipose tissue by regulating lipid metabolic pathways. Moreover, the steroid metabolic pathway can affect the numbers of T cells in adipose tissue and play dual roles in the regulation of immune functions in adipose tissue, i.e., up-regulation suppressing immunity and down-regulation promoting immunity. In addition, we discovered a potential mechanism for regulating cholesterol metabolism (miR-29a/c-3p-*HMGCR* network) in adipose tissue and evaluated the potential application value of circulating miR-29a/c-3p. This study can provide a directional reference for further investigating immune regulatory functions of lipid metabolism in adipose tissue.

## Figures and Tables

**Table 1:** Primers used in this study.

Primer Name	Sequence (5’–3’)
*β-actin* forward	TATGTGCAAGGCCGGTTTC
*β-actin* reverse	TTCAGGGTCAGGATACCTCTTT
*ATGL* forward	CTGATACCTCCAACTTTGCGTG
*ATGL* reverse	GTAGAGGTTGCGAAGGTTGAAT
*CPT1A* forward	AGTCCGGCCACTTATGAATGAT
*CPT1A* reverse	ATTATTGGTCCACGCCCTC
*TCRá* forward	TTTGGAAATGTGCCTTATCACGG
*TCRá* reverse	TCATGTTTGTTCTCGGATGTTGC
*HMGCR* forward	CTCAGGAGCGAGGAGTGTCTAT
*HMGCR* reverse	GTATAGTGGTCCTGCTACGCCT
miR-29a/c-3p RT	CTCAACTGGTGTCGTGGAGTCGGCAATTCAGTTGAGAACCGATT
*U6* RT	AACGCTTCACGAATTTGCGT
miR-29a/c-3p forward	ACACTCCAGCTGGGTAGCACCATTTGAAA
miR-29a/c-3p reverse	TGGTGTCGTGGAGTCG
*U6* forward	CTCGCTTCGGCAGCACA
*U6* reverse	AACGCTTCACGAATTTGCGT

## Data Availability

The datasets are available from the corresponding author on reasonable request.

## Abbreviations

ATGL	adipose triglyceride lipase
CPT1A	carnitine palmitoyltransferase 1A
HMGCR	3-hydroxy-3-methylglutaryl-CoA reductase
qRT-PCR	quantitative real-time fluorescence PCR
Dex	dexamethasone
WAT	white adipose tissue
TME	tumor microenvironment
FFAs	free fatty acids
FABP	fatty acid binding protein
AT	Adipose tissue
TRM	resident memory T cell
SIIS	stress-induced immunosuppression
TCRα	T-cell receptor α
Acetyl-CoA	acetyl coenzyme A
TCA	tricarboxylic acid
